# Profiling tumour heterogeneity through circulating tumour DNA in patients with pancreatic cancer

**DOI:** 10.18632/oncotarget.20250

**Published:** 2017-08-14

**Authors:** Patricia Adamo, Caroline M Cowley, Christopher P Neal, Vilas Mistry, Karen Page, Ashley R Dennison, John Isherwood, Robert Hastings, JinLi Luo, David A Moore, Pringle J Howard, Martins L Miguel, Catrin Pritchard, Margaret Manson, Jacqui A Shaw

**Affiliations:** ^1^ Department of Cancer Studies, University of Leicester, Robert Kilpatrick Clinical Sciences Building, Leicester Royal Infirmary, Leicester, UK; ^2^ Department of Hepatobiliary and Pancreatic Surgery, Leicester General Hospital, Leicester, UK; ^3^ Cancer Research UK Leicester Centre, University of Leicester, Leicester, UK; ^4^ MRC Toxicology Unit, Leicester, UK

**Keywords:** ctDNA, cfDNA, pancreatic ductal adenocarcinomas, KRAS, liquid biopsy

## Abstract

The majority of pancreatic ductal adenocarcinomas (PDAC) are diagnosed late so that surgery is rarely curative. Earlier detection could significantly increase the likelihood of successful treatment and improve survival. The aim of the study was to provide proof of principle that point mutations in key cancer genes can be identified by sequencing circulating free DNA (cfDNA) and that this could be used to detect early PDACs and potentially, premalignant lesions, to help target early effective treatment. Targeted next generation sequencing (tNGS) analysis of mutation hotspots in 50 cancer genes was conducted in 26 patients with PDAC, 14 patients with chronic pancreatitis (CP) and 12 healthy controls with *KRAS* status validated by digital droplet PCR. A higher median level of total cfDNA was observed in patients with PDAC (585 ng/ml) compared to either patients with CP (300 ng/ml) or healthy controls (175 ng/ml). PDAC tissue showed wide mutational heterogeneity, whereas *KRAS* was the most commonly mutated gene in cfDNA of patients with PDAC and was significantly associated with a poor disease specific survival (p=0.018). This study demonstrates that tNGS of cfDNA is feasible to characterise the circulating genomic profile in PDAC and that driver mutations in *KRAS* have prognostic value but cannot currently be used to detect early emergence of disease. Importantly, monitoring total cfDNA levels may have utility in individuals “at risk” and warrants further investigation.

## INTRODUCTION

Pancreatic cancer is the eleventh most common cancer in the UK, accounting for 3% of all newly diagnosed cancers, but is the fifth leading cause of cancer-related death [1]. The incidence increases with age and worldwide the five-year survival rate is ≤7% [2]. Late presentation is a feature of the disease with only 15-20% of cancers being resectable at diagnosis due to the location of the pancreas deep within the abdomen [3]. Due to the considerably shorter median survival of locally advanced (9-11 months) and metastatic disease (6-8 months) compared to resectable disease (13-20 months), there is a growing consensus that these should be viewed as prognostically (and possibly biologically) different disease entities [4]. Pancreatic ductal adenocarcinoma (PDAC) is the most common type of pancreatic cancer, representing around 80% of all cases. These tumours often have a high stromal content creating a “fortress-like” hypovascular barrier that is thought to impair the delivery of chemotherapeutics and promote aggressive neoplastic cell behaviour [5]. Both the location and composition of the tumours explains why imaging techniques often fail to detect early lesions and have difficulty distinguishing benign from malignant disease.

The causes of pancreatic cancer are unknown but numerous studies have identified genetic and modifiable risk factors including chronic pancreatitis (CP) [6]. A meta-analysis demonstrated a relative risk of 13.3 for CP patients developing PDAC but with a ten to twenty year lag between the incidences of pancreatitis and pancreatic malignancy [7]. Therefore, it is still undetermined whether CP is a precursor disease to PDAC. A progression model has been proposed starting with the precursor disease of pancreatic intraepithelial neoplasia (PanIN) and developing in a step-wise manner through the successive accumulation of specific mutations [8]. This includes activation of the oncogene *KRAS* at a relatively early stage, inactivation of the tumour suppressor gene *CDKN2A* at an intermediate stage and at a late stage, inactivation of the tumour suppressor genes *TP53* and *SMAD4* [8]. However, Notta *et al* [9] have suggested that the switch in certain cases can be a result of simultaneous mutations in these genetic drivers and it has not been confirmed that *KRAS* is the gatekeeper required for neoplasia despite its occurrence in 70-90% of PDAC tissue.

Currently the diagnosis of pancreatic cancer involves tissue biopsy but this is not always possible and can give false negatives due in part to the high stromal content of many pancreatic cancers. Moreover, tissue biopsy is expensive and difficult to perform, and can result in trauma to the patients [10]. A further disadvantage is that a tissue biopsy can only give site-specific information due to sampling techniques and may prevent a more complete overview of the tumour; this is particularly pertinent when considering the high genetic heterogeneity of pancreatic cancer if mutational profiling was to be conducted clinically [11]. An alternative approach under current investigation is the use of the tumour derived fraction of circulating free DNA (cfDNA) termed circulating tumour DNA (ctDNA) as a surrogate or “liquid biopsy” of the tumour. ctDNA originates from tumour cells undergoing apoptosis or necrosis during the natural development of the cancer from the primary tumour or metastatic lesions and therefore is considered to be less impacted by intratumour heterogeneity than a single tissue biopsy [12]. This cfDNA/ctDNA has a short half-life (approx. 2 hours) allowing for the evaluation of tumour changes in hours rather than weeks to months [13] making it ideal for progression and treatment monitoring [14]. In addition, liquid biopsies are cheaper, faster, more comfortable for patients and easy to repeat. ctDNA has been detected previously in pancreatic cancer, but with a sensitivity ranging from 26-100% due to studies differing in the platforms used and the genetic markers examined [3].

The detection of circulating tumour-derived biomarkers in blood could provide a relatively non-invasive approach for earlier detection of pancreatic ductal adenocarcinoma (PDAC). Here we aimed to provide proof of principle that point mutations in key cancer genes can be identified by targeted amplicon sequencing of cfDNA isolated from blood plasma, which, with appropriate validation, may detect early PDACs and, potentially, premalignant lesions, helping target effective treatment. To achieve this we profiled and compared the cfDNA from patients with PDAC to patients with CP and healthy controls. Where available, these profiles were compared to matched FFPE tumour tissue.

## RESULTS

### Panel validation

We first tested the sensitivity of the Cancer Hotspot Panel v2, which comprises 207 amplicons covering approximately 2,800 COSMIC mutations from 50 cancer genes, to detect hot spot mutations in *KRAS, SMAD4, CDKN2A* and *TP53* [15, 16]. Using 1 ng, 5 ng and 10 ng DNA derived from three pancreatic cell lines (BxPC-3, PANC-1, MIA PaCa-2) [10], expected COSMIC mutations were detected in each cell line and the mutation frequency was consistent across the three DNA concentrations (Table 1).

**Table 1:** Validation of the Cancer Hotspot Panel v2 by detecting hotspot mutations in four commonly mutated genes

Gene mutation; amino acid (Variant Allele Frequency)												
Cell line	KRAS			TP53			SMAD4			CDKN2A		
	10 ng	5 ng	1 ng	10 ng	5 ng	1 ng	10 ng	5 ng	1 ng	10 ng	5 ng	1 ng
**BxPC-3**	Wild Type			c.659A>G; Y220C (100%)	c.659A>G; Y220C (100%)	c.659A>G; Y220C (100%)	Homozygous deletion			Homozygous deletion		
**PANC-1**	c.35G>A; p.G12D (70.5%)	c.35G>A; p.G12D (70.2%)	c.35G>A; p.G12D (72.2%)	c.818G>A; R273H (100%)	c.818G>A; R273H (100%)	c.818G>A; R273H (100%)	Wild Type			Homozygous deletion		
**MIA PaCa-2**	c.34G>T; p.G12C (100%)	c.34G>T; p.G12C (100%)	c.34G>T; p.G12C (100%)	c.742C>T; R248W (100%)	c.742C>T; R248W (100%)	c.742C>T; R248W (100%)	Wild Type			Homozygous deletion		

Allele frequency shown in brackets where appropriate.

### Total cfDNA levels are higher in PDAC than controls

**Figure 1:**
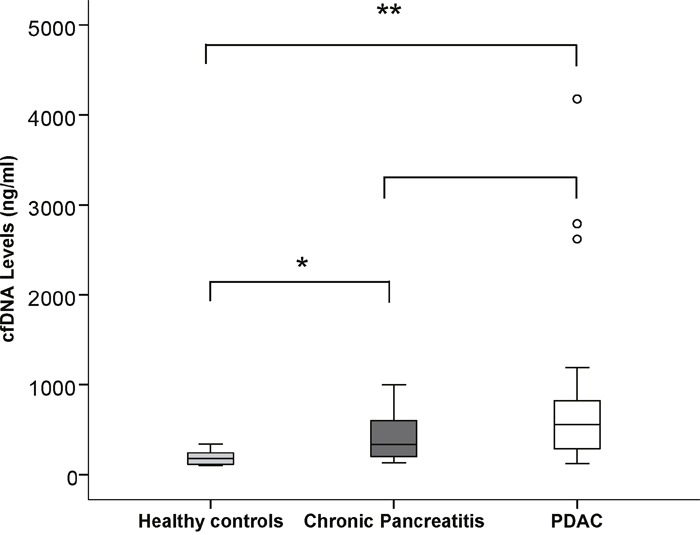
Stem and Leaf plot demonstrating the differences in cfDNA levels between healthy controls, CP and PDAC patients A significantly higher expression was noted in CP patients compared to healthy controls and in PDAC patients compared to healthy controls but not between CP patients and PDAC. ^*^ p<0.05, ^**^ p<0.001.

We quantified total cfDNA levels in 12 healthy controls, 14 patients with CP and 26 patients with PDAC (resectable PDAC n=6, primary non-resectable PDAC n=5 and metastatic non-resectable PDAC n=15). Table 2 shows the clinicopathological criteria for these groups with a full summary per patient in Supplementary Table 1. The total cfDNA levels ranged from a median of 175 ng/ml for health controls to 300 ng/ml for CP and 585 ng/ml for PDAC. Levels were significantly different between healthy controls and both patients with CP (p=0.003) and PDAC (p=<0.001) but not between patients with CP and PDAC (p=0.086) (Figure 1). There were 3 patients with CP (35, 81 and 88) who died during the course of the study from a non-cancer related death who had the highest total cfDNA levels overall (600, 780 and 1000 ng/ml respectively).

**Table 2:** Clinicopathological characteristics of cfDNA patients

	Healthy Volunteers N (%)	Chronic Pancreatitis N (%)	PDAC N (%)
**Total number of patients**	12	14	26
**cfDNA (ng/ml)**			
Median	175	300	585
Range	100-340	130-1000	120-4180
**Age (years)**			
Median	55	56	69
Range	40-83	19-71	40-84
**Sex**			
Male	2 (17)	10 (72)	16 (62)
Female	10 (83)	3 (21)	10 (38)
Unknown	0 (0)	1 (7)	0 (0)
**Ethnicity**			
Caucasian	na	na	25 (96)
Other			1 (4)
**TNM Staging**			
I/II	-	-	7 (27)
III	-	-	2 (8)
IV	-	-	16 (61)
Unknown	-	-	1 (4)
**Site of Tumour**			
Head	-	-	19 (73)
Body, Tail, Neck	-	-	6 (23)
Unknown	-	-	1 (4)
**Ca19.9 levels (U/ml)**			
≤ 37	-	-	3 (11)
> 37	-	-	15 (58)
Not Tested	-	-	8 (31)
**Survival (days)**			
Median	na	1482	148
Range		101-1579	17-1460
**Disease Specific Survival**			
Alive	na	11 (79)	1 (4)
Cancer death		0 (0)	25 (96)
Non Cancer death		3 (21)	0 (0)

na; data not available, -; not applicable to patient cohort.

In PDAC, the different subgroups (resectable PDAC, primary non-resectable PDAC and metastatic non-resectable PDAC) were not significantly different from one another and total cfDNA levels were not associated with disease specific survival (p=0.273, Hazard Ratio 1.000, 95% Confidence Interval 1.0-1.0), with the presence of distant metastasis (p=0.517) or with Ca19.9 levels (p=0.301).

### Mutation profiling and prognostic value of somatic mutations in cfDNA

Mutation profiling was conducted using tNGS on cfDNA from 12 healthy controls, 14 patients with CP and 26 patients with PDAC. *KRAS* mutation status was validated by ddPCR in all cases with sufficient remaining cfDNA after tNGS. A detectable mutation was found in 1 healthy control (*JAK2* p.V617F mutation, variant allelic fraction (VAF) =8%) and in 1 CP patient, multiple mutations were detected (in *STK11, PDGFRA, KIT* and *KDR*) suggesting genomic instability (Supplementary Table 2).

From the 26 PDAC cases, only 1 of the 6 patients with primary resectable PDAC had a mutation in their cfDNA (*KRAS* p.G12D mutation; VAF=8.7%). None of the primary non-resectable PDAC cases had any detectable mutations (Patient 76 was not amplifiable by tNGS but was found to be wild type for *KRAS* during validation of cases by ddPCR) (Table 3 and Supplementary Table 3). The 15 patients with metastatic non-resectable PDAC had disease in the peritoneum, lung, liver or a combination (Supplementary Table 1). Six patients (40%) had either a *KRAS* p.G12R (67%) mutation or a *KRAS* p.G12V (33%) mutation in cfDNA with variant allele frequencies ranging from 1.4 to 62.9% (Table 3 and Supplementary Table 3). Two of the detected *KRAS* mutations (patients 14 and 18) had variants called below the tNGS quality score applied to this study but were confirmed by ddPCR. Only 1 patient (31) had additional mutations to *KRAS* in cfDNA, in *TP53* p.R248Q (VAF=17.4%) and *SMAD4* p.P356R (VAF=13%). The VAFs were at similar frequencies to the *KRAS* p.G12V mutation (VAF=16.1%) suggesting these were derived from a single predominant clone.

**Table 3:** Summary of the mutations identified in the cfDNA of PDAC patients by tNGS

	Patient ID	cfDNA (ng/ml)	Gene	Mutation	Variant Allele Frequency (%)	Coverage	COSMIC
**Resectable PDAC**	63	540	KRAS^*^	WT	--	606	--
	69	800	KRAS^*^	WT	--	482	--
	72	250	KRAS	WT	--	507	--
	92	220	KRAS	WT	--	980	--
	102	840	KRAS^*^	WT	--	1448	--
	104	450	KRAS^*^	**p.G12D**	**8.7**	**473**	**COSM521**
**Primary Non-Resectable PDAC**	16	232	KRAS	WT	--	853	--
	19	300	KRAS^*^	WT	--	2040	--
	34	1190	KRAS^*^	WT	--	1257	--
	76^**^	354	KRAS	WT	na	na	na
	80	610	KRAS^*^	WT	--	464	-
**Metastatic Non-Resectable PDAC**	12	120	KRAS	WT	--	431	--
	13	270	KRAS^*^	WT	--	456	--
	14	710	KRAS^*^	***p.G12V***	**1.4**	**1094**	**COSM520**
	15	700	KRAS^*^	WT	--	2000	--
	18	700	KRAS^*^	***p.G12R***	**1.5**	**834**	**COSM518**
	21	2790	KRAS^*^	**p.G12R**	**1.8**	**925**	**COSM518**
	24	300	KRAS	WT	--	1155	--
	26	4180	KRAS^*^	WT	--	330	--
	27	2620	KRAS^*^	**p.G12R**	**62.9**	**705**	**COSM518**
	31	430	KRAS^*^	**p.G12V**	**16.1**	**996**	**COSM520**
			SMAD4	p.P356R	13.0	1642	COSM339351
			TP53	p.R248Q	17.4	3857	COSM10662
	41	1150	KRAS^*^	WT	--	388	--
	53	970	**KRAS**	**p.G12R**	**23.8**	**408**	**COSM518**
	68	280	KRAS^*^	WT	--	1338	--
	73	710	KRAS^*^	WT	--	930	--
	91	560	KRAS^*^	WT	--	597	--

--; not relevant as wild type, ^*^; validated by ddPCR, ^**^; identified by ddPCR validation, na; data not available.

Comparing the cfDNA *KRAS* mutation status to clinicopathological criteria for all PDAC cases (Table 4), there was no significant association between *KRAS* mutations and distant metastasis (p=0.190). The presence of *KRAS* mutations in cfDNA was significantly associated with the tumour being sited at the body, tail or neck of the pancreas (p=0.015) compared to the head of the pancreas. *KRAS* status did not associate with TNM stage (p=0.109) or Ca19.9 levels (p=1.00).

**Table 4:** Association of KRAS in the cfDNA of PDAC patients with clinicopathological criteria

Variable	KRAS		
	NoN (%)	YesN (%)	*p* value
**Total number of PDAC patients**	19	7	
**Site of Tumour**			
Head	17 (90)	2 (29)	**0.015**
Body, Tail, Neck	2 (10)	4 (57)	
Unknown	0 (0)	1 (14)	
**Metastasis**			
No	9 (47)	1 (14)	0.190
Yes	10 (53)	6 (86)	
**TNM Staging**			
I/II	7 (37)	0 (0)	0.109
III	2 (10)	0 (0)	
IV	10 (53)	6 (86)	
Unknown	0 (0)	1 (14)	
**Ca19.9 levels (U/ml)**			
≤ 37	2 (10)	1 (14)	1.000
> 37	11 (58)	4 (57)	
Unknown	6 (32)	2 (29)	

**Figure 2:**
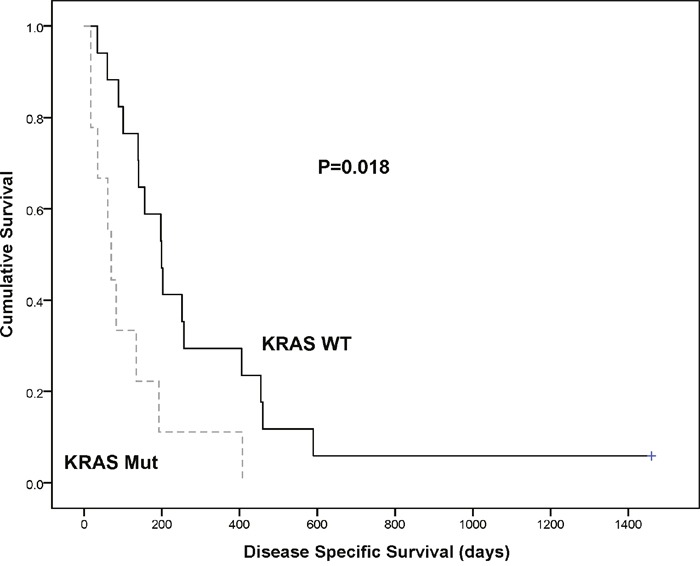
Kaplan Meier survival curve demonstrating *KRAS* mutation (dotted line) significantly associates with a poorer disease specific survival vs *KRAS* wild type (solid line) when detected in cfDNA of PDAC patients (p=0. 018)

The presence of *KRAS* mutations in the cfDNA of patients with PDAC was associated with poor disease specific survival (p=0.018, hazard ratio=2.889, 95% confidence interval (CI) =1.2-7.3) (Figure 2 and Table 5). The median survival time was 60 days for *KRAS* positive PDAC and 197 days for *KRAS* negative PDAC. In general, the presence of metastasis (p=0.013), the site of the tumour (p<0.001) and the TNM stage (p=0.015) in the PDAC cases associated with disease specific survival but Ca19.9 levels (p=0.620), gender (p=0.833) and age (p=0.655) did not (Table 5).

**Table 5:** Log rank analysis of *KRAS* mutation status in the cfDNA of PDAC patients and relevant clinicopathological criteria with disease specific survival

Variable	Disease Specific Survival					
	N (%)	Median Survival (days)	X^2^ value	*p* value	Hazard Ratio	95% Confidence Interval
**Total number of PDAC patients**	26	148				
**KRAS**						
WT	19 (73)	197	5.589	**0.018**	2.889	1.2 - 7.3
Mutant	7 (27)	60				
**Metastasis**						
No	10 (39)	255	7.007	**0.013**	3.399	1.3 - 8.9
Yes	16 (61)	111				
**Site of Tumour**						
Head	19 (73)	199	12.592	**<0.001**	11.266	3.1 - 41.6
Body, Tail, Neck	6 (23)	35				
Unknown	1 (4)	60				
**TNM Staging**						
I/II	7 (27)	257	6.980	**0.015**	1.995	1.1 - 3.5
III	2 (8)	421				
IV	16 (61)	111				
Unknown	1 (4)	60				
**Ca19.9 levels (U/ml)**						
≤ 37	3 (11)	455	0.261	0.620	1.385	0.4 - 5.0
> 37	15 (58)	140				
Unknown	8 (31)	145				
**Gender**						
Male	16 (61)	195	0.044	0.833	1.092	0.5 - 2.5
Female	10 (39)	117				
**Age (years)**						
< 65	7 (27)	139	0.200	0.655	0.818	0.3 - 2.0
≥ 65	19 (73)	192				

### Mutation profiling of matched tumour tissue

Eleven of the cfDNA patients (4 resectable PDAC, 2 primary non-resectable PDAC and 5 metastatic non-resectable PDAC) had matching primary tumour tissue. The tissue was analysed by tNGS (Supplementary Table 4) and *KRAS* mutation status validated by ddPCR. No mutations were detected in the cfDNA of these patients. Eight out of the 11 tumour tissues (73%) had a *KRAS* mutation with p.G12D (4/8, 50%) being the predominant mutation, followed by p.G12V (3/8, 38%). Two of the *KRAS* mutations (patients 34 and 15) had a quality score below the tNGS threshold for variant calling in this study, but were validated by ddPCR. A further *KRAS* mutation (in case 72) was identified by ddPCR but not by tNGS as it was below the limit of detection for the panel (see Supplementary Table 5) (1/8, 12%).

Additional heterogeneous mutations were detected in the tissue of 5 patients. Of note was a *CDKN2A* p.R80^*^ mutation in patient 41 (average VAF=31.1% based on two tissue regions, see Supplementary Table 5), detected at a similar allele frequency to the *KRAS* mutation (average VAF=31.2% based on two tissue regions see Supplementary Table 5). The remaining mutations were detected at a lower VAF suggesting that these were sub-clonal to mutations in *KRAS*. There was no pattern to the types of mutations or prevalence between the different sub groups of PDAC.

### Mutational heterogeneity is seen in tumour tissues

In 8 patients with PDAC, multiple tumour tissue regions were available (Supplementary Table 5) and these were analysed by tNGS to assess intratumoural heterogeneity. For *KRAS*, only two patients had a mutation detected in all regions (patient 36, *KRAS* p.G12V and patient 41, *KRAS* p.G12D). In 3 patients (patients 100, 102, 51), *KRAS* was detected by tNGS in some regions but was below the limit of detection for the panel in other regions and therefore only identified by ddPCR.

In patient 40, region 40.2 had a *KRAS* p.G12C variant called below the tNGS quality score cut off but was validated by ddPCR. In region 40.1, no *KRAS* mutation could be detected by tNGS or ddPCR (the number of positive mutation droplets for ddPCR was below the cut off applied for the study). In patient 68, all three regions differed. Region 68.1 had a *KRAS* p.G12D detected by tNGS, in region 68.4 *KRAS* was below the limit of detection for tNGS and only identified through ddPCR validation and in region 68.3 there was no detectable *KRAS* by either method. In patient 72, the *KRAS* mutations were only detected during ddPCR validation. This demonstrates the mutational heterogeneity in different regions of pancreatic tumour tissues.

There was even greater heterogeneity with gene variants other than *KRAS* across all patients as the majority were solely found in only one of the tissue regions. The exceptions were *CDKN2A* p.R80^*^ in patient 41, which was found in both tissue regions and the deletions in *EGFR* p.E746_A750delELREA, which were detected in 2 out of 3 regions in patient 51. Patient 45 was the only case that had two different *KRAS* mutations detectable by tNGS in the same tumour tissue region, p.G12D (VAF=17%) and p.G12C (VAF=15.4%).

## DISCUSSION

In this study we aimed to provide proof of principle that point mutations in key cancer genes could be identified by sequencing cfDNA and provide a relatively non-invasive approach to detecting early PDACs and potentially, premalignant lesions, to help target early effective treatment. Only 1 CP patient had detectable somatic mutations in cfDNA, and of note was the mutation in the tumour suppressor gene, *STK11*, that encodes for a serine/threonine kinase, a known genetic risk factor for PDAC [6]. Therefore, it may have been of value to monitor this patient through serial blood samples although the patient did not develop PDAC during the course of the study. Previous studies have detected *KRAS* mutations in the cfDNA of patients with CP, which were not detectable in this study. However, these studies followed the patients for a period of time (ranging from 6 to 64 months), and none of the patients developed pancreatic cancer [17, 18]. Due to the difficulty in diagnosing PDAC especially in patients with CP, a separate study [7] looked at the relationship between Ca19.9 combined with *KRAS* mutation and suggested that in a CP patient with normal Ca19.9 levels and no *KRAS* mutations, the diagnosis of pancreatic cancer can be excluded with almost certainty. A study to validate this finding is warranted due to the inadequacy of standard imaging and tissue biopsies and we would also recommend inclusion of the preneoplastic lesions, PanINs 1a/b, 2 and 3 [8]. The *JAK2* p.V617F mutation found in the cfDNA of one healthy control is a pathogenic risk factor but is not directly related to PDAC [19]. The lack of significance between the total cfDNA levels in CP patients and PDAC (p=0.086) may be explained by the inflammatory nature of chronic pancreatitis: Inflammation causes cell death, with ensuing release of cfDNA into the blood stream [20].

In the cfDNA of patients with PDAC, *KRAS* was the most frequently detected mutation. Castells *et al* were the first to detect circulating mutant *KRAS* genes in plasma from patients with PDAC in 1999 [17] and mutations in *KRAS* are reported to be among the earliest to occur during carcinogenesis [21, 22] and therefore may be founder mutations [8]. Overall 27% (7/26) of patients with PDAC had a detectable *KRAS* mutation in plasma cfDNA by tNGS, which is in the range reported in other studies (27-81%) (reviewed in [23]). This range of *KRAS* mutation detection is mainly due to the following factors: different stages of pancreatic cancer examined (i.e. late stage unresectable 81% [24] vs earlier stage primary operable 27% [17]) and differences in methods and sensitivity of these methods. This study was designed as a proof of concept, to include all the different stages of PDAC, and to assess an up to date, diagnostically relevant technique, tNGS.

We found that the presence of mutant *KRAS* was associated with a poor disease specific survival (p=0.018) (60 days vs 197 days). This has been observed in previous studies [17, 21, 23, 25] and interestingly Hadano *et al* [26] found that the presence of mutant *KRAS* was only significantly associated with a poor prognosis if it was detected in the plasma samples but not if the expression was only analysed in the matching tissue samples. Another study found no statistical significance [18] but as stated there is a large variability in previous studies in patient groups, technical approaches and sample preparation. In this study, the presence of *KRAS* mutations was also significantly associated with the tumour being sited at the body, tail or neck of the pancreas (p=0.015), known to have an inferior survival outcome compared to the tumour being sited in the head of the pancreas [27].

In the cfDNA of a metastatic non-resectable PDAC patient, a *SMAD4* (VAF=13%) and *TP53* mutation (VAF=17.4%) was detected at a similar frequency to the *KRAS* mutation (VAF=16.1%) but there was no *CDK2NA* mutation. This differs to the progression model proposed by Hruban *et al* [8], however, the model is based on progression from PanIN and it is not known if the PDAC lesions in this study originated from PanIN or other preneoplastic conditions.

In the 11 patients for whom matching cfDNA and tumour tissue samples were available, no mutations were detected in the cfDNA despite detection of mutations in the tissue. PDAC is known to be a hypovascular tumour and it is thought that due to this, DNA may not be released into the circulation as readily, especially in the earlier stages of tumour development [21]. This may explain the low number of patients with mutations in cfDNA overall and compounding this, PDAC cancers have a low median tumour cellularity of around 30% [22] due to a high stromal content [5]. The sequencing depth at the *KRAS* locus also varies due to the efficiency of the PCR reaction at that locus and because both cfDNA and FFPE samples can vary in quality. The more fragmented a sample is, the more difficult it is to amplify.

In the PDAC tissue samples, a lot of intertumoral heterogeneity was observed. A previous comprehensive genetic analysis of 24 pancreatic cancers [11], showed that the genetic basis of pancreatic cancer is extremely complex and heterogeneous with an average of 63 relevant genetic abnormalities per tumour, which was organized into 12 functional cancer relevant pathways.

We also observed intratumoral heterogeneity between different regions within the same tumour, which is an important considerations for any future tissue based biomarker analysis as only taking samples from one part of the tumour is likely to miss this. In later stages of the disease, sampling purely at the primary tumour site will also not be able to take into account the intermetastatic heterogeneity between metastatic sites within the patient, which would affect the ability to personalise treatment. With the additional complication of the inaccessibility of pancreatic tumours, the less invasive liquid biopsy approach is still a viable alternative to overcome these limitations in PDAC.

From the important mutations known to occur in PDAC [28], *TP53* was the most frequently observed mutation in the tissue in association with *KRAS* but was generally at a lower frequency. Only one metastatic case had a detectable *CDK2NA* mutation. Further somatic mutations of note were the *EGFR* deletions. Erlotinib is the only targeted agent that has demonstrated a statistically significant effect on overall survival when combined with Gemcitabine in advanced pancreatic cancer [29]. Detection of deletions via tNGS is not optimal and therefore future studies would use ddPCR validation. Unfortunately all of the cases with an *EGFR* mutation also had a *KRAS* mutation and it has been observed in colorectal cancer (although inconclusive in NSCLC) that a high frequency of *KRAS* mutations limits the benefits of EGFR inhibitors [30] as it leaves the KRAS protein turned “on” and signalling within the cancer cell continues regardless of the fact that the EGFR receptor is blocked.

To compare the *KRAS* mutation frequencies to other studies, all PDAC patients were taken into account (15 cfDNA only, 11 cfDNA and tissue, 7 tissue only), therefore overall 27% (7/26) of PDAC had a detectable *KRAS* mutation in plasma with 78% percent (14/18) in tissue. Castells *et al* [17] reported a similar ratio with 72% (28/39) in the primary tumour and 32% (9/28) showing an identical alteration in the corresponding plasma. From the 20 PDAC patients with known *KRAS* mutation types, 35% had a p.G12D mutation (7/20), 35% a p.G12V (7/20), 20% a p.G12R (4/20) and 15% a p.G12C (3/20) representing the four most common *KRAS* mutations at codon 12 (patient 45 had a p.G12C and p.G12D mutation). In Europe and China, the most common alterations in the *KRAS* oncogene are reported to be in the order of p.G12D (c.35G>A), p.G12V (c.35G>T), p.G12R (c.34G>C) and p.G12C (c.34G>T), whereas in the USA the order has been reported to be p.G12D (c.35G>A), p.G12V (c.35G>T), p.G12C (c.34G>T) and p.G12R (c.34G>C) (reviewed in [23] and [31]). In a separate study of a Japanese cohort, p.G12V had the highest prevalence (37.3%), correlating with a shorter survival [32] and found to be more invasive *in vitro* [33] but this relationship has differed in other studies [34]. We also found one case that had two *KRAS* mutations coexisting at similar frequencies (p.G12D VAF=17% and p.G12C VAF=15.4%) something that has been mainly observed in pancreatic cancer with associated PanIN [35].

In conclusion, we demonstrated mutational heterogeneity in primary tumour tissue of patients with PDAC, and found circulating tumour derived DNA in 27% of patients based on detection of mutations in the *KRAS* gene. These results demonstrate that point mutations in key cancer genes can be identified by tNGS of cfDNA. Moreover, monitoring the levels of cfDNA in PDAC patients for driver mutations in the *KRAS* gene has prognostic value warranting a larger study. Unfortunately, this study was not able to help detect early PDACs or premalignant lesions of pancreatic cancer but since this study, there have been more advances in tNGS, allowing for better detection of low frequency variants, and combined with the recommendations for standardization of study cohorts and extraction methods, this may help with future validation of cfDNA biomarkers.

## MATERIALS AND METHODS

### Patients and samples

A total of 59 patients were analysed for the study; 51 had amplifiable cfDNA from plasma and 18 had amplifiable DNA from formalin fixed paraffin embedded (FFPE) tissue samples. Full details including clinicopathological characteristics are presented in Supplementary Table 1. Blood sample collection was conducted in accordance with the Declaration of Helsinki and all samples were taken prior to treatment. The study protocol was approved by the Leicestershire, Rutland and Northamptonshire Research Ethics Committee (University Hospitals of Leicester NHS Trust; REC reference number: 7176). All eligible patients during the course of the study gave written informed consent prior to participation. Blood was taken by venepuncture into K2 EDTA-containing collection tubes (BD Biosciences, San Jose, CA, USA) and processed as described previously [36]. The median age for all patients was 66 with a range of 19-84. The median survival was 1482 days for patients with CP and 150 days for patients with PDAC.

### Cell culture

Pancreatic cancer cell lines (PANC-1, BxPC-3 and MIA PaCa-2) obtained from ATCC (Manassas, Virginia, USA) were used for assay development and validation. Cells were cultured in DMEM + 10% FBS (Sigma-Aldrich, St Louis, Mo, USA). Prior to extraction cells were trypsinised and washed in 1X PBS (Sigma). Cells were pelleted and stored at −80°C. Cell pellets were reconstituted in 200μl of 1X PBS before extraction.

### Extraction and quantitation of DNA

cfDNA was extracted from plasma using the QIAamp Circulating Nucleic Acid kit (Qiagen, Hilden, Germany) as per the manufacturer's instructions. DNA was extracted from pancreatic cancer cell lines using the DNA blood mini kit (Qiagen) according to the manufacturer's instructions. FFPE tissue blocks with matching H&E were retrieved from the histopathology archive and reviewed by a consultant Histopathologist. DNA from FFPE samples was extracted using the GeneRead DNA FFPE Kit (Qiagen). DNA was quantified using the Qubit 2.0 fluorometer according to manufacturers’ instructions.

### Targeted next generation sequencing (tNGS)

Reactions were set up using the Ion Ampliseq^TM^ Cancer Hotspot Panel V2 (4475346, Thermo Fisher Scientific, Waltham, Mass., USA) using 10 ng of cfDNA or 20 ng FFPE DNA. Samples were sequenced on the Ion Personal Genome Machine (Ion PGM^TM^) using Ion 316^TM^ v2 chips according to manufacturer's instructions. Sequencing data was analysed through the Torrent Suite^TM^ v4.2.0. Reads were aligned against the human genome (hg19) using Alignment v4.0-r77189 and variants called using the coverageAnalysis v4.0-r77897 and variantCaller v4.0-r76860, respectively [10]. COSMIC IDs were obtained using COSMIC v76 (http://cancer.sanger.ac.uk/cosmic, last accessed 2^nd^ May 2016). All mutations with a quality score below 20 were omitted and all variants detected in the first or last 10 bases of an amplicon were omitted as likely mispriming events [37]. All variants detected were also manually confirmed across all samples using the Integrated Genomics Viewer 5.01. The mutant variant allele frequency (VAF) was calculated as the proportion of total reads at a site, which contained the variant allele.

### Droplet digital PCR (ddPCR)

Droplet digital PCR was used to validate the *KRAS* mutational status of patient samples where possible. 5 ng of cfDNA and 10 ng of FFPE DNA was used in conjunction with Bio-Rad's ddPCR^TM^ *KRAS* G12/G13 Screening kit according to manufacturers’ instructions (dHsaMDV2510586, dHsaMDV2510584, dHsaMDV2510596, dHsaMDV2510590, dHsaMDV25 10588, dHsaMDV2510592, dHsaMDV2510598, Bio-Rad, Hercules, Calif., USA) using a Bio-Rad QX200 digital droplet PCR system as previously described [38]. For all assays, no template controls were run to determine lack of contamination. All assays with ≥4 mutant droplets were considered positive for the *KRAS* mutation. Raw fluorescence amplitude was analysed using the Quantasoft version 1.6.6.0320 software to obtain the fractional abundance (FA) of mutant DNA alleles to total (mutant plus wild type) DNA alleles.

### Statistical analysis

SPSS version 22 statistical software package was used. A stem and leaf plot was constructed to demonstrate the difference in cfDNA levels between the different groups of patients and the Independent Samples Mann-Whitney U test. For PDAC patients, disease specific survival was calculated from the date of the blood sample until 15^th^ September 2015 when any remaining survivors were censored. The Mann Whitney U test was used to examine the levels of cfDNA between PDAC patients with and without distant metastasis and to the Ca19.9 levels. The association between the *KRAS* status and metastasis was assessed using a Fisher's exact test due to there being less than five cases in some cells. Survival analysis comparing age, gender or *KRAS* status with disease specific survival for the PDAC patients was conducted using the log rank test and Kaplan-Meier curves. Hazard ratios and 95% confidence intervals where determined using Cox regression analysis as was the relationship between cfDNA levels and disease specific survival. The correlation between *KRAS* and distant metastasis was confirmed using Spearman rank-order.

### Abbreviations

PDAC: Pancreatic Ductal Adenocarcinoma
cfDNA: Circulating free DNA
ctDNA: Circulating tumour DNA
CP: Chronic Pancreatitis
tNGS: Targeted Next Generation Sequencing
ddPCR: Droplet Digital PCR
PanIN: Pancreatic Intraepithelial Neoplasia

## SUPPLEMENTARY MATERIALS TABLES












