# Supplementary material for: Profiling tumour heterogeneity through circulating tumour DNA in patients with pancreatic cancer
Source: Oncotarget. 2017 Aug 14;8(50):87221–33. doi: 10.18632/oncotarget.20250 (PMC5675628; doi:10.18632/oncotarget.20250)
Supplement: Supplementary file 1 [file oncotarget-08-87221-s001.pdf]

Profiling tumour heterogeneity through circulating tumour DNA in patients with pancreatic cancer

SUPPLEMENTARY MATERIALS

Supplementary Table 1: Sample summary and clinicopathological criteria for all patients

See Supplementary File 1

Supplementary Table 2: Mutations identified in the cfDNA of healthy controls and CP patients by tNGS

See Supplementary File 2

Supplementary Table 3: Mutations identified in the cfDNA of PDAC patients by tNGS

See Supplementary File 3

Supplementary Table 4: Summary of the mutations identified in the matching cfDNA and tissue of PDAC patients by tNGS

See Supplementary File 4

Supplementary Table 5: Mutations identified in different tissue regions of PDAC patients by tNGS

See Supplementary File 5
